# Effect of Replacing Corn with Rice on Growth Performance, Meat Quality, Gut Microbiota and Metabolites in Growing–Finishing Pigs

**DOI:** 10.3390/ani16010012

**Published:** 2025-12-19

**Authors:** Xiaolin Wu, Qinqun Jiang, Hong Hu, Qi Han, Xihong Zhou

**Affiliations:** 1 College of Animal Science and Technology, Hunan Agricultural University, Changsha 410128, China; v2637227@gmail.com (X.W.); huhong7777777@126.com (H.H.); 2 Institute of Subtropical Agriculture, Chinese Academy of Sciences, Changsha 410125, China; 3 Pingdingshan Academy of Agricultural Sciences, Pingdingshan 467000, China; jiaweiflying@163.com

**Keywords:** cellulase, growth performance, gut microbiota, paddy rice, pork quality

## Abstract

This study aimed to investigate the effects of partially replacing corn with rice in the diet and supplementing with cellulase on growth performance, pork quality, and gut microbiota of growing–finishing pigs. A total of 64 healthy pigs (68.03 ± 1.59 kg) were randomly assigned into four groups: CON (basal diet), ASE (basal diet + 20,000 IU/g cellulase), RICE (the basal diet replaced 50% of corn with rice), RASE (the basal diet replaced 50% of corn with rice + 20,000 IU/g cellulase). The results indicated that dietary rice and cellulase, either individually or in combination, had no significant effects (*p* > 0.05) on the FBW, ADG, ADFI, and F/G of pigs. However, the dietary rice markedly increased the intramuscular fat (IMF) content (*p* < 0.05). Furthermore, gut microbiota profiling indicated that *Papillibacte* were enriched in the RICE group and positively associated with IMF content. Metabolomic analysis revealed substantial differences in fecal metabolites among the treatment groups. The RICE group exhibited reduced levels of Zygadenine, Carpaine, and Rhodioloside E, which were negatively correlated with both IMF content and *Papillibacter*. In conclusion, dietary rice can promote IMF deposition by modulating gut microbiota and host metabolism, without compromising the growth performance of pigs.

## Simple Summary

Rice, a widely cultivated grain, has emerged as a promising alternative to corn, the primary energy source in conventional pig diets. However, the potential effects of replacing corn with rice on pig growth performance have not been fully assessed, and its influence on pork quality and underlying mechanisms remains unclear. This study examined the impact of replacing 50% of dietary corn with rice, with or without added cellulase, in growing–finishing pigs. We evaluated growth performance, gut microbiota, fecal metabolites, and pork quality. The results showed that replacing half of the corn with rice did not impair growth performance. Instead, it significantly increased intramuscular fat (IMF), a key indicator of meat quality. Rice-based diets also reshaped the gut microbiota and metabolite profiles. Notably, the abundance of *Papillibacter* increased and was positively associated with IMF levels, while several metabolites—including Zygadenine, Carpaine, and Rhodioloside E—decreased. These metabolites were negatively related to both *Papillibacter* and IMF, suggesting that modifications in the microbiota–metabolite axis may contribute to enhanced fat deposition. Overall, incorporating rice into pig diets improved pork quality by increasing IMF without compromising growth, supporting the use of rice as a promising feed ingredient.

## 1. Introduction

With the rapid advancements in technology, genetics, and management in the livestock industry, coupled with the growing demand for meat, there has been an increasing emphasis on diversifying and optimizing feed resources. In this context, the effective utilization of locally available feed ingredients to enhance meat quality and support healthy livestock production has emerged as a key research focus. Corn, a staple in conventional animal feed, is subject to availability constraints due to its reliance on specific geographic regions and climatic conditions [1]. Moreover, its price is highly volatile, driven by fluctuations in market supply and demand. Consequently, the search for alternative feed ingredients with high nutritional value and cost-efficiency has become a pressing priority.

Rice is a major cereal crop whose by-products (e.g., broken rice, rice bran) are important and economically viable feed ingredients, especially in rice-producing regions such as Asia [2,3]. According to data from the Food and Agriculture Organization of the United Nations, Asia dominates global rice production, accounting for approximately 90% of the world’s rice output by 2023, with a total of 716.78 million metric tons. This provides a solid foundation for the large-scale utilization of rice by-products as animal feed. Compared to corn, rice contains a significantly higher crude fiber content—exceeding 8.5%, which is more than four times that of corn [4]. However, its digestible energy and crude protein contents for pigs are approximately 11.62 MJ/kg and 7.8%, respectively—both around 10% lower than those of corn. In terms of essential amino acid composition, rice contains slightly lower levels of histidine and leucine compared to corn, but has a higher lysine, threonine, and isoleucine content [5].

The incorporation of rice into pig diets is primarily limited by its high crude fiber content and the presence of various anti-nutritional factors [6]. These anti-nutritional components—mainly non-starch polysaccharides (NSPs), resistant starch, phytic acid, uronic acid, trypsin inhibitors, and lectins—are predominantly concentrated in the rice hull and bran layers [5]. Specifically, NSPs (e.g., arabinoxylans and β-glucans) increase digesta viscosity in the intestinal tract, thereby reducing the contact efficiency between endogenous enzymes and nutrients and impeding nutrient diffusion across the intestinal epithelium [7]. Phytic acid chelates divalent cations (e.g., Fe^2+^, Zn^2+^, Ca^2+^) and forms insoluble complexes with proteins, hindering their absorption and utilization [8]. As a result, the direct use of rice in animal feed can lead to reduced nutrient digestibility and limited utilization. To enhance the nutritional value of rice in feed formulations, supplementation with feed enzymes or enzymatic pretreatment technologies have emerged as a promising approach. Previous research suggested that the supplementation of phytase and xylanase in animal feed improved the nutritional value and utilization of feed ingredients by degrading anti-nutritional factors [9]. Enzymatic microbial pretreatment involves the application of specific enzymes and microorganisms to degrade fiber and anti-nutritional factors, thereby improving the digestibility of feed ingredients [10]. This process has been shown to increase feed intake and promote better growth performance in animals. Moreover, enzymatic microbial treatment may also improve meat quality by enhancing flavor and tenderness [11].

Therefore, we hypothesize that partially replacing corn with rice in pig diets, coupled with enzyme supplementation, could present a strategy to address feed cost pressures while potentially enhancing meat quality. The present study aims to evaluate the effects of partial replacement of corn with rice and supplementation with or without cellulase in basal diets on the growth performance, intestinal microbiota composition, and meat quality of fattening pigs. The findings are expected to provide a scientific basis for the development of corn alternatives in animal nutrition and to support the improvement of meat quality through the utilization of locally sourced specialty feed ingredients.

## 2. Materials and Methods

### 2.1. Experimental Design

An experiment was conducted in Yuanjiang City, Hunan Province, using 86 healthy male pigs (Landrace × Yorkshire × Duroc) with an average body weight of 68.03 ± 1.59 kg. All pigs were maintained in pens fitted with slatted floors, stainless-steel feeders, and nipple drinkers. Pens with comparable average body weight, stocked at 3–4 pigs per pen, were randomly assigned to different dietary treatments following a 2 × 2 factorial arrangement integrated with a randomized complete block design. The two factors were diet type (corn-based basal diet or 50% corn-replaced rice diet) and cellulase supplementation (0 or 20,000 IU/g cellulase), forming four treatment groups: CON, basal diet; RICE, basal diet in which 50% of the corn was replaced by paddy rice; ASE, basal diet supplemented with 20,000 IU/g cellulase; RASE, basal diet with 50% corn replaced by paddy rice and supplemented with 20,000 IU/g cellulase. The block factors of the randomized complete block design were the initial body weight of the pens and the spatial distribution of pen space within the barn. Initially, the pens were grouped based on average initial weight, and then the positions within the pen were assigned to eliminate the effects of environmental heterogeneity, such as ventilation and temperature.

Each group consisted of six replicate pens, with three to four pigs per pen. Feed and water were available ad libitum for the entire experiment. The paddy rice used in this study was sourced from a commercial rice mill in Yuanjiang, China, and ground to a fine particle size (approximately 0.5 mm) using a 2 mm screen. As shown in Table 1, all diets were pelleted and formulated to meet or exceed the nutrient requirements for growing–finishing pigs, as recommended by the NRC (2012) [12]. The basal diet was primarily composed of corn and soybean meal. Chromium oxide served as an indigestible marker for determining the apparent total tract digestibility (ATTD) of nutrients, as outlined in a previous study [7].

### 2.2. Chemical Analyses of Diet Composition

All chemical analyses were performed in accordance with the methods of the Association of Official Analytical Chemists (AOAC, 2005) [13]. Each prepared sample was analyzed in duplicate for the following components: dry matter (DM, Method 934.01), ether extract (EE, Method 920.39), crude fiber (CF, Method 985.29), crude ash (CA, Method 942.05), and starch (Method 979.10). An Ankom 2000 Fiber Analyzer (Macedon, NY, USA) was used to assess neutral detergent fiber (NDF) and acid detergent fiber (ADF) contents. Nitrogen content was quantified with a KDN-103 automatic Kjeldahl nitrogen analyzer (Shanghai, China), and crude protein (CP) was derived by applying a conversion factor of 6.25 to the nitrogen values (Method 990.03). Gross energy (GE) was obtained using an MP-C 2000 bomb calorimeter (Mingpeng Technology, Changsha, China), and an atomic absorption spectrophotometer (Biotek, Rochester, NY, USA) was employed to measure chromium content. The composition and analyzed nutritional content of the experimental diets are provided in Table 1.

### 2.3. Performance Evaluation and Sample Collection

The pigs were slaughtered until they reached a body weight of approximately 130 kg. Body weight and feed intake were monitored throughout the experimental period to calculate average daily gain (ADG), average daily feed intake (ADFI), and the feed-to-gain ratio (F/G). To determine nutrient digestibility, fresh fecal samples were collected at the conclusion of the trial. For sample collection, six pigs from each treatment group (one pig per pen) were randomly selected and slaughtered. Samples of serum, *longissimus dorsi* muscle, and intestinal contents were collected for further analysis of relevant physiological and nutritional parameters.

### 2.4. Nutritional Apparent Digestibility Analysis

Moisture content in feed and raw materials was measured according to the national standard GB/T 6435-2006 [14] “Determination of Moisture and Other Volatile Matter in Feed”. Dry matter content in feces and urine was also determined. Gross energy in feed and feces was measured using an oxygen bomb calorimeter. Crude protein in raw materials and feed was determined according to the national standard GB/T 6432-1994 [15] “Method for Crude Protein Determination in Feed”. Nitrogen content in digesta was measured by the Kjeldahl method. Crude ash in raw materials and feed was determined following the national standard GB/T 6438-2007 [16]“Determination of Crude Ash in Feed”. Crude fiber was analyzed following GB/T 6434-2006 [17]. Neutral detergent fiber and acid detergent fiber were determined according to GB/T 20806-2006 [18] and NY/T 1459-2007 [19], respectively. Other components were analyzed in accordance with the corresponding national standards, specifically including ether extract (GB/T 6433-2006 [20], Determination of Ether Extract in Feed) and chromium (GB/T 13088-2006 [21], Determination of Chromium in Feed).

(1) Calculation of Digestible Energy, Metabolizable Energy, and Digestibility of Feed

Apparent Digestible Energy (DE, kcal/kg) of the diet was calculated as:

DE = (Gross Energy Intake − Gross Energy in feces)/Feed Intake (kg)

Apparent Metabolizable Energy (ME, kcal/kg) of the diet was calculated as:

ME = (Gross Energy Intake − Gross Energy in feces − Gross Energy in urine)/Feed Intake (kg)

Apparent Total Tract Digestibility (ATTD, %) of gross energy was calculated as:

ATTD = (Gross Energy Intake − Gross Energy in feces)/Gross Energy Intake × 100

(2) Calculation Method for Apparent Total Tract Digestibility (ATTD) of Nutrients

Apparent total tract digestibility of nutrients in the diet (ATTD, %) was calculated as:

ATTD = (Nutrient Intake − Nutrient Content in feces)/Nutrient Intake × 100

Apparent total tract digestibility of nutrients in the test ingredient (ATTD, %) was calculated as:

ATTD = (ATTD of nutrient in diet − (100% − X%) × ATTD of nutrient in basal diet)/X%

where X% is the proportion of the test ingredient replacing the basal diet on an energy basis.

(3) Calculation of Energy Values of Test Ingredients Using the Difference Method

Energy value = (Energy value of test diet − (100% − X%) × Energy value of basal diet)/X%

where (X%) is the percentage of the test ingredient replacing the basal diet on an energy basis; energy values include digestible energy (DE) and metabolizable energy (ME), expressed in kcal/kg.

### 2.5. Serum Biochemical Index

Blood samples were collected from all pigs at slaughter to assess lactate concentration and serum creatine kinase (CK) activity, both of which are commonly used as indicators of physical fatigue [22,23]. The evaluation of these physiological stress markers was intended to verify that all pigs were in a comparable physiological state at the time of slaughter, thereby minimizing the influence of pre-slaughter stress as a confounding factor in meat quality assessment. In addition, serum levels of alanine aminotransferase (ALT) and aspartate aminotransferase (AST) were measured to evaluate liver function and overall metabolic status.

### 2.6. Meat Quality

Samples of the *longissimus dorsi* muscle were collected, and meat quality was evaluated in accordance with the Technical Regulation for Pork Quality Measurement (NY/T 821-2019 [24]). The following parameters were measured: meat color (lightness L*, redness a*, yellowness b*), muscle pH, marbling score, drip loss, storage loss, tenderness, and cooking yield. Meat tenderness was assessed based on the Determination of Meat Tenderness-Shear Force Measurement Method (NY/T 1180-2006 [25]). Specifically, the *longissimus dorsi* muscle samples (size: 1.0 cm × 1.0 cm × 3.0 cm, with the long axis parallel to the muscle fiber direction) were taken from the left carcass at 24 h post-slaughter and equilibrated to room temperature (25 °C). Shear force was measured using a TA-XT Plus texture analyzer (Stable Micro Systems, Godalming, UK) equipped with a V-shaped blade probe (HDP/BS).

### 2.7. RNA Extraction and Quantification

Gene expression was determined by real-time PCR. Total RNA was isolated from muscle tissues using RNAiso Plus (Takara, Dalian, China) and subsequently reverse-transcribed into cDNA using the PrimeScript RT reagent kit with gDNA Eraser (Takara, Dalian, China), following the manufacturer’s protocols. Amplification was performed on a LightCycler480 system (Roche, Basel, Switzerland). The relative mRNA expression of each target gene was normalized to the expression of the housekeeping gene β-actin. Table S1 lists the primer sequences used in this study.

### 2.8. High-Throughput 16S rRNA Sequencing

Genomic DNA was isolated from DLY pig fecal samples with a QIAamp DNA Stool Mini Kit (Qiagen, Hilden, Germany). The DNA concentration and purity of extracted DNA were assessed on 1% agarose gels. The DNA was diluted to a concentration of 1 ng/mL using sterile water. The primers 515F: 5′-GTGCCAGCMGCCGCGGTAA-3′ and 806R 5′-GGACTACHVGGGTWTCTAAT-3′ were performed for amplifying the V3–V4 hypervariable variable region of the bacterial 16S rRNA gene. Single amplifications reactions were carried out in a 25 μL volume using 50 ng of template DNA per reaction. The PCR protocol consisted of an initial denaturation step at 94 °C for 4 min, followed by 30 cycles of denaturation at 94 °C for 30 s, annealing at 54 °C for 30 s, and extension at 72 °C for 30 s, with a final extension at 72 °C for 5 min. The resulting PCR products were normalized to equimolar concentrations, pooled, and subjected to sequencing on the Illumina HiSeq2500 platform (San Diego, CA, USA). We performed bioinformatics analyses with QIIME 1.9.1 to evaluate α-diversity (Simpson, Chao1, and Shannon indices), β-diversity, and bacterial abundance.

### 2.9. Untargeted Metabolomics Analysis

Metabolomics analysis was performed by Novogene Co., Ltd. (Beijing, China). Each tissue sample (100 mg) was pulverized under liquid nitrogen. The resulting homogenate was re-suspended in pre-chilled 80% methanol and subjected to thorough vortex mixing. Subsequently, the samples were maintained on ice for 5 min followed by centrifuged at 15,000× *g*, 4 °C for 20 min. The extract was diluted with mass spectrometry-grade water to adjust the methanol content of the solution to 53%, the mixture was centrifuged again, and the resulting supernatant was then analyzed by UHPLC–MS/MS analysis. Separation was performed employing a Hypesil Gold C18 column (0.2 mL/min). Mobile phase A consisted of 0.1% formic acid (positive mode) or 5 mM ammonium acetate (negative mode), while mobile phase B was methanol. The solvent gradient underwent the following program: 2% B was held for 1.5 min, increased linearly from 2% to 100% B over 3 min, maintained at 100% B for 10 min, decreased to 2% B over 10.1 min, with a final re-equilibrated at 2% B for 12 min. Parameters for the mass spectrometer were as follows: a spray voltage of 3.5 kV; sheath gas at 35 psi; a capillary temperature of 320 °C; an S-lens RF level of 60; an auxiliary gas flow of 10 L/min; and an auxiliary gas heater temperature of 350 °C. Differential metabolites were identified based on a thresholds of VIP (Variable Importance in Projection) > 1, *p*-value < 0.05, and |FC| ≥ 1.5. For data structure visualization and differential feature display, Principal Component Analysis PCA (PCA) and volcano plots were generated utilizing the metaX platform and the R package ggplot2 (version 4.3.2), respectively.

### 2.10. Measurement of Odor-Active Volatile Compounds Volatile Compound

The present analysis followed a previously described method with minor adjustments [26]. Briefly, each *longissimus dorsi* muscle sample (3 g) was precisely weighed into a 20 mL headspace vial. Subsequently, 2 μL of 50 μg/mL n-Pentadecane-d32 (internal standard) was introduced, then supplemented with 5 mL of saturated sodium chloride solution. The vial was promptly sealed in preparation for metabolomic profiling based on HS-SPME-GC-MS. The analysis was performed by Majorbio Bio-Pharm Technology Co., Ltd. (Shanghai, China). Volatile sampling was carried out using an SPME Arrow fiber, with analysis performed on a TRACE 1610 GC system interfaced with an Orbitrap Exploris MS (Thermo Fisher Scientific, Waltham, MA, USA) and a TriPlus RSH SMART autosampler. The extraction conditions were: incubation at 80 °C for 20 min, extraction at 80 °C for 10 min, fiber head aging at 240 °C, pre-desorb and post-desorb fiber conditioning for 2 min each, desorption time of 1 min, and a 20 mL sample bottle.

The sample was introduced into the GC–MS system via split injection (split ratio 10:1) with an injection volume of 1 μL. Compound separation was achieved using a VF-WAXms capillary column (25 m × 0.25 mm × 0.2 μm) under a constant helium flow (99.999% purity, 1 mL/min). The injector was maintained at 240 °C. The GC column temperature was set as follows: initial hold at 40 °C, ramped to 120 °C at 8 °C/min, then increased to 230 °C at 20 °C/min, and finally held for 4.5 min, yielding a total run time of 20 min. The mass spectrometric was operated in electron impact (EI) ionization at 70 eV with an ion source temperature set of 250 °C. The full scan mass range was set to *m*/*z* 35–500 with a resolving power of 30,000 (FWHM). Raw data from GC/MS detection was preprocessed with Compound Discovery 3.3 SP3 software. Volatile metabolites were annotated through alignment of mass spectra and calibrated retention indices against two reference databases: NIST 2023 and the Thermo Scientific GC-Orbitrap Flavor and Fragrances v1.0 library. Semi-quantitative analysis of volatiles was performed by normalizing chromatographic peak areas against the concentration of the internal standard.

### 2.11. Statistical Analysis

All data are presented as mean ± SEM. Differences among the groups were compared were conducted by the general linear model (GLM) for two-way statistical analysis of variance and significance tests in SPSS 27.0 software (SPSS Inc., Chicago, IL, USA). The fixed effects of the statistical model included diet type, cellulase supplementation, and their interaction. Since the initial body weight and rearing environment of the pens were strictly standardized prior to the experiment, the heterogeneity between pens was negligible, and hence, no random effects were implemented. The differences between groups were assessed using Tukey’s multiple comparison method for post hoc analysis. All figures were generated using GraphPad Prism 8.0.2 software. *p* <0.05 was considered as statistical significance, whereas 0.05 ≤ *p* < 0.1 was considered as indicative of a trend.

## 3. Results

### 3.1. Growth Performance

As shown in Table 2, the final body weight (FBW), ADG, ADFI, and F/G of growing–finishing pigs were not affected by the inclusion of rice or the addition of cellulase (*p* > 0.05).

### 3.2. Apparent Digestibility

The effects of dietary rice and cellulase supplementation on the apparent digestibility of nutrients in growing–finishing pigs are presented in Table 3. In comparison to the control group, dietary rice decreased the apparent digestibility of crude protein, crude fiber, and gross energy (*p* < 0.05). However, cellulase supplementation had no effect on the apparent digestibility of CP, CF, and GE compared with the control group (*p* > 0.05). No significant interaction between rice and cellulase was observed for the apparent digestibility of CP, CF, and GE (*p* > 0.05).

### 3.3. Blood Biochemistry

As shown in Table 4, compared with the control group, the rice diet increased lactate levels (*p* < 0.05). Cellulase supplementation resulted in lower ALT and lactate levels compared to the control group (*p* < 0.01). Additionally, the CK levels did not differ significantly across the four treatment groups (*p* > 0.05), suggesting that the dietary interventions did not induce chronic muscle damage or systemic inflammation. Significant interactions between the rice diet and cellulase supplementation were observed for ALT and AST levels.

### 3.4. Pork Quality

The effects of rice and cellulase on pork quality in growing–finishing pigs are shown in Table 5. The rice group significantly increased IMF content (*p* < 0.01) compared to the control group, with no alterations observed in backfat thickness (BFT), drip loss (DL), pH at 45 min (pH45), pH at 24 h (pH24), or color (*p* > 0.05). Additionally, supplementation with cellulase had no significant effect on pork quality relative to the control (*p* > 0.05). There were also no significant interactive effects between the rice diet and cellulase on BFT, DL, pH45, pH24, color, or IMF content.

### 3.5. Expression of Lipid Metabolism-Related Genes

The impacts of rice and cellulase on lipid metabolism-related genes in *longissimus dorsi* muscle of growing–finishing pigs are shown in Table 6. Compared with control group, dietary rice downregulated the expression of *PPARα* (*p* < 0.05) and tended to enhance the expression of *CD36* (0.05 < *p* < 0.1). The cellulase supplementation downregulated the expression of *PPARα*, *SREBP2*, and *DGAT2* (*p* < 0.05). And there were no significant interactive effects between the rice diet and cellulase on the *PPARα*, *SREBP1*, *SREBP2*, *LXRβ*, *CD36*, *DGAT1* and *DGAT2*.

### 3.6. Intestinal Microbiota Composition

**Figure 1:**
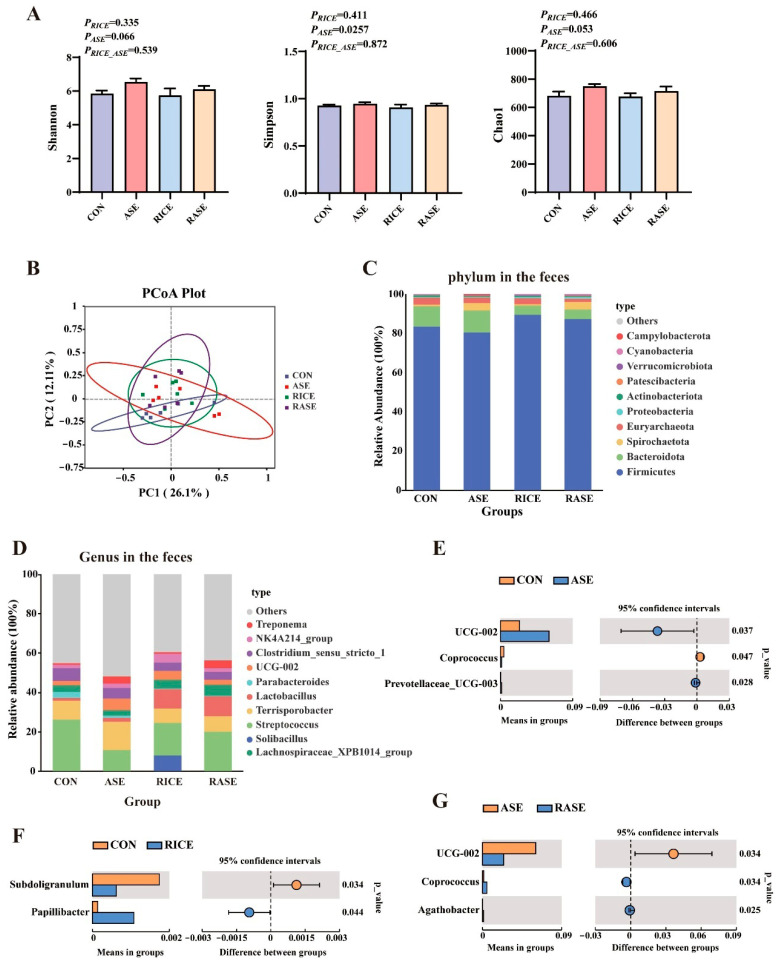
Effect of rice and cellulase on the intestinal microbiota composition in growing–finishing pigs. (**A**) The Shannon, Simpson, and Chao diversity indexes. (**B**) Principal coordinate analysis (PCoA) cluster diagram. (**C**) Abundance Phyla in the feces (Top 10). (**D**) Abundance genera in the feces (Top 10). (**E**) Differential genera in ASE vs. CON. (**F**) Differential genera in RICE vs. CON. (**G**) Differential genera in RASE vs. ASE.

As shown in Figure 1, cellulase supplementation enhanced bacterial richness and alpha-diversity, illustrated by increasing trends in the Shannon and Chao1 indices (0.05 < *p* < 0.1) relative to the control group (Figure 1A). However, no significant interaction between rice and cellulase, nor any main effect of rice on the alpha-diversity indices, was observed (Figure 1A). Additionally, principal coordinates analysis (PCoA) showed no clear separation in microbiota composition among the different groups (Figure 1B).

Next, we examined the microbiota composition of growing–finishing pigs at the phylum and species levels. The top 10 most abundant phyla were as follows: Firmicutes, Bacteroidota, Spirochaetota, Euryarchaeota, Proteobacteria, Actinobacteriota, Patescibacteria, Verrucomicrobiota, Cyanobacteria, and Campylobacterota (Figure 1C). Compared with the control group, the rice diet markedly enhanced the abundance of Firmicutes and Cyanobacteria (*p* < 0.05), while decreasing the abundance of Bacteroidota (*p* < 0.05) (Table S2). Cellulase supplementation markedly increased the abundance of Spirochaetota (*p* < 0.05) (Table S2). No significant interactions between rice and cellulase were observed in the relative abundance of the top 10 microbial phyla.

At the genus level, the relative abundance of the top 10 bacterial genera is shown in Figure 1D and Table S3, among which a notable increase in *Lactobacillus* was observed in pigs fed the rice-based diet. Relative to the control group, the ASE group significantly resulted in a significant increase in *UCG_002* (*p* < 0.05) and *Prevotellaceae_UCG-003*, whereas a decrease was observed in *Coprococcus* (Figure 1E). The rice diet significantly enhanced the abundance of *Papillibacte* (*p* < 0.05) (Figure 1F). Moreover, the RASE group exhibited a significant reduction in *UCG-002* (*p* < 0.05) relative to the ASE group, while conversely enriching the abundances of *Coprococcus* and *Agathobacter* (*p* < 0.05) (Figure 1G).

### 3.7. Metabolite Compositions of Feces

**Figure 2:**
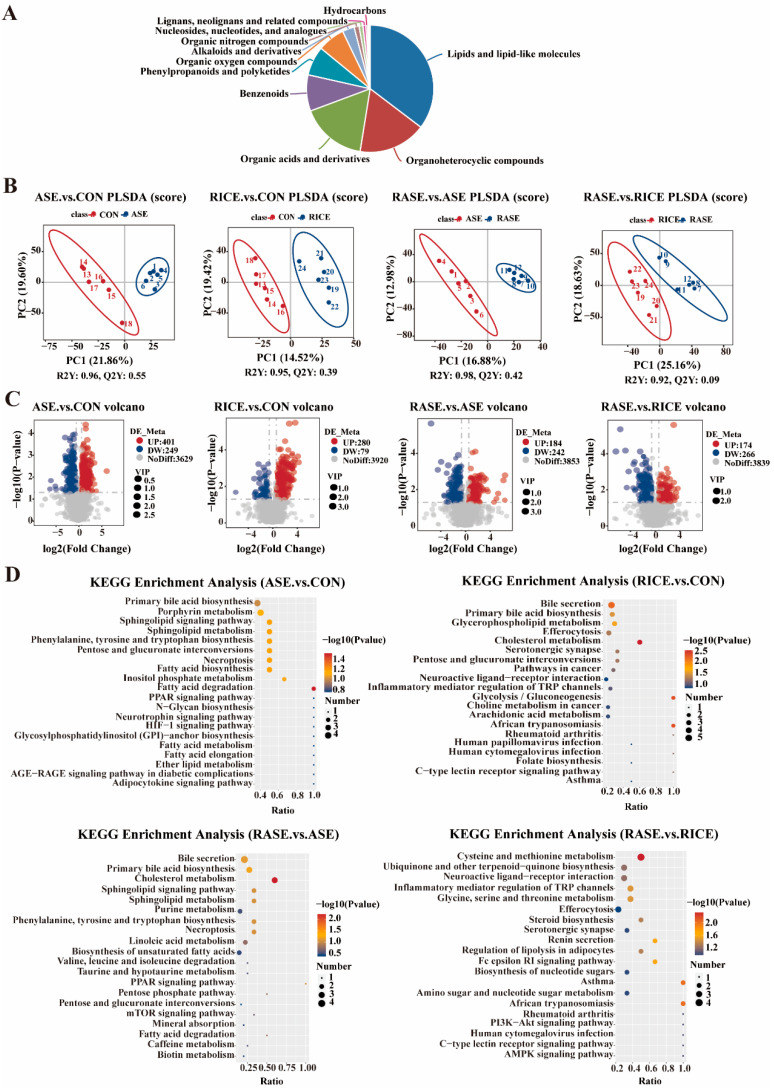
Effect of rice and cellulase on the metabolomic profile in the feces of growing–finishing pigs. (**A**) Percentage of different metabolites class. (**B**) Scatter plots of partial least squares-discriminant analysis (PLS-DA). (**C**) Volcano plot (metabolites that are upregulated are depicted as red circles, downregulated metabolites as blue circles, and metabolites with no significant difference are represented by gray circles). (**D**) Enrichment of the top 20 KEGG pathways.

To investigate the effects of rice and cellulase on growing–finishing pigs, the metabolite composition of feces was analyzed using LC-MS/MS. In total, 4179 metabolites were detected across the four groups. These metabolites were categorized into 18 groups, with the top three being lipids and lipid-like molecules (35.34%), organoheterocyclic compounds (17.21%), and organic acids and derivatives (16.77%) (Figure 2A). Distinct clustering of the groups was observed in the PLS-DA score plot, indicating significant metabolic disparities (Figure 2B).

Next, differential metabolites screened with the thresholds of VIP > 1, fold change (FC) > 1.5 or < 0.67, and *p*-value < 0.05. The volcano plot (Figure 2C) showed that 650 differential metabolites were found between the ASE and CON groups (401 upregulated, 249 downregulated). Additionally, 359 differential metabolites were screened between the RICE and CON groups (280 upregulated, 79 downregulated), 326 between the RASE and ASE groups (184 upregulated, 242 downregulated), and 440 between the RASE and RICE groups (174 upregulated, 266 downregulated).

KEGG pathway enrichment analysis (Figure 2D) showed that in the ASE vs. CON comparison, the differential metabolites were mainly enriched in fatty acid degradation, pentose and glucuronate interconversions, and sphingolipid metabolism. In the RICE vs. CON comparison, the most enriched pathways included cholesterol metabolism, glycolysis/gluconeogenesis, glycerophospholipid metabolism, primary bile acid biosynthesis, and bile secretion. Pathway analysis of the RASE vs. ASE comparison identified multiple enriched processes, with the top hits including cholesterol metabolism, primary bile acid biosynthesis, and bile secretion. Finally, in the RASE vs. RICE comparison, significant enrichment was observed in cysteine and methionine metabolism, glycine, serine, and threonine metabolism, and the regulation of lipolysis in adipocytes.

### 3.8. Correlation Analysis of the Gut Microbiota, Fecal Metabolites and IMF

**Figure 3:**
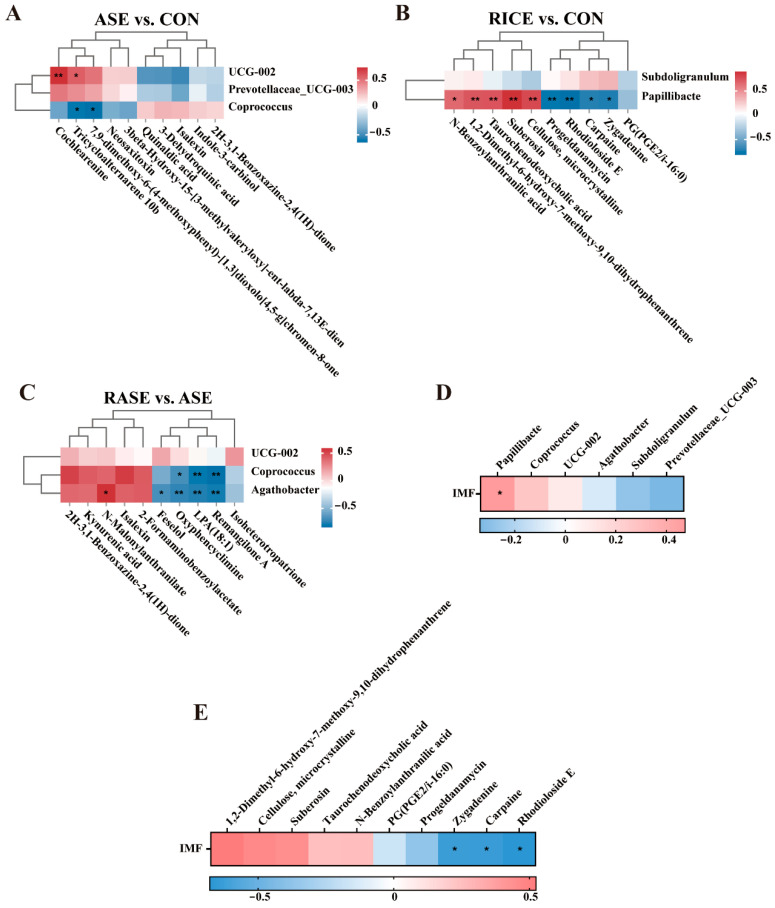
Spearman correlation analysis. (**A**) Microbiota and metabolites in ASE vs. CON. (**B**) Microbiota and metabolites in RICE vs. CON. (**C**) Microbiota and metabolites in RASE vs. CON. (**D**) IMF content and differential microbiota. (**E**) IMF content and differential metabolites. Statistical significance is denoted by asterisks: * *p* < 0.05, ** *p* < 0.01. Key correlation pairs and their corresponding r/*p* values are as follows: (1) *Papillibacter* vs. IMF content: r = 0.474, *p* = 0.022; (2) *Papillibacter* vs. Zygadenine: r = −0.718, *p* = 0.013; *Papillibacter* vs. Carpaine: r = −0.709, *p* = 0.015; *Papillibacter* vs. Rhodioloside E: r = −0.800, *p* = 0.003; (3) IMF content vs. Zygadenine: r = −0.636, *p* = 0.026; IMF content vs. Carpaine: r = −0.643, *p* = 0.024; IMF content vs. Rhodioloside E: r = −0.678, *p* = 0.015.

Spearman correlation analyses were performed to investigate the potential relationships between the differential gut microbiota at the genus level and the ten most significantly altered fecal metabolites. In the ASE vs. CON comparison, marked positive correlations existed between *UCG-002* and Cochlearenine, as well as Tricycloalternarene 10b. Negative correlations were found between *Coprococcus* and Tricycloalternarene 10b (Figure 3A). In the RICE vs. CON comparison, *Papillibacter* showed strong associations with various metabolites, being positively correlated with N-Benzoylanthranilic acid, 1,2-Dimethyl-6-hydroxy-7-methoxy-9,10-dihydrophenanthrene, Taurochenodeoxycholic acid, Suberosin, Cellulose, and microcrystalline, while negatively correlated with Zygadenine, Carpaine, Rhodioloside E, and Progeldanamycin (Figure 3B). In the RASE vs. ASE comparison, *Coprococcus* was negatively correlated with Remangilone A, LPA (18:1), and Oxyphencyclimine. Furthermore, *Agathobacter* exhibited negative correlations with Remangilone A, LPA (18:1), Oxyphencyclimine, and Feselol, but a positive correlation with N-Malonylanthranilate (Figure 3C).

Furthermore, Spearman correlation analysis revealed that the *Papillibacter* was positively correlated with IMF content in pigs (Figure 3D). Notably, the metabolites (Zygadenine, Carpaine, and Rhodioloside E) that correlated with *Papillibacter* also showed a negative association with IMF content (Figure 3E). Collectively, our findings suggested that *Papillibacter* appears to regulate IMF deposition by acting to decrease the levels of the Zygadenine, Carpaine, and Rhodioloside E.

### 3.9. Volatile Flavor Compounds in the Longissimus Dorsi Muscle

**Figure 4:**
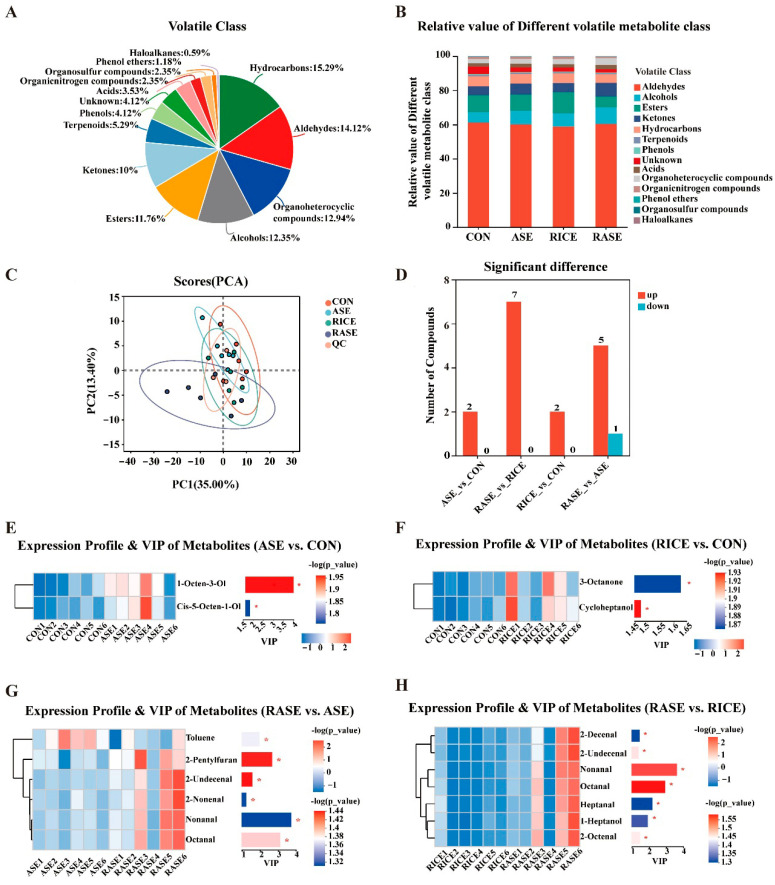
Differential VOCs identification in *longissimus dorsi* muscles. (**A**) Percentage of different volatile metabolites class. (**B**) Relative contents and types of volatile compound. (**C**) PCA scores are based on VOCs from LD muscles of 24 samples. (**D**) Statistical chart of Vocs differences among different groups. (**E**) Heatmap and VIP value of differential VOCs between CON and ASE group. (**F**) Heatmap and VIP value of differential VOCs between CON and RICE group. (**G**) Heatmap and VIP value of differential VOCs between RASE and ASE group. (**H**) Heatmap and VIP value of differential VOCs between RASE and RICE group. * *p* < 0.05, was considered as statistical significance.

A total of 170 volatile compounds were identified across 24 porcine skeletal muscles. These volatile flavor compounds (VOCs) were classified into 14 major categories: 26 hydrocarbons (15.29%), 24 aldehydes (14.12%), 22 organoheterocyclic compounds (12.94%), 21 alcohols (12.35%), 20 esters (11.76%), 17 ketones (10%), 9 terpenoids (5.29%), 6 acids (3.53%), 4 organic nitrogen compounds (2.35%), 4 organosulfur compounds (2.35%), 2 phenol ethers (1.18%), 1 haloalkane (0.59%), and others (4.12%) (Figure 4A). The results, as shown in Figure 4B, indicate the relative contents and types of volatile compounds. Aldehydes, alcohols, esters, ketones, and heterocyclic compounds were the primary volatile flavor substances in pork. The most notable difference was observed in alcohol content: the relative content of alcohols in the CON group (6.13%) was lower than in the ASE (7.98%) and RICE (7.79%) groups, while the RASE group exhibited the highest alcohol content (9.76%) (Table S4). Additionally, the relative content of esters in the RICE group (12.28%) was higher than in the CON (9.82%), ASE (9.46%), and RASE (6.25%) groups (Table S4). However, principal component analysis (PCA) revealed substantial overlap among the four groups, with no clear clustering pattern based on treatment (Figure 4C).

Further analysis identified differential VOCs among the groups (Figure 4D). The contents of 1-Octen-3-ol and Cis-5-Octen-1-ol were significantly higher in the ASE group than in the CON group (Figure 4E). Additionally, the 3-Octanone and Cycloheptanol contents in the RICE group were significantly higher than in the CON group (Figure 4F). The RASE group showed significantly higher levels of 2-Pentylfuran, 2-Undecenal, 2-Nonenal, Nonanal, and Octanal compared to the ASE group, while its Toluene content was significantly lower (Figure 4G). Moreover, compared to the RICE group, the RASE group exhibited significantly higher levels of 2-Decenal, 2-Undecenal, Nonanal, Octanal, Heptanal, 1-Heptanol, and 2-Octenal (Figure 4H). Compounds with a relative odor activity value (ROAV) > 1 were considered the primary flavor contributors. Based on the analysis of flavor compound differences and ROAV (Table S5), 1-Octen-3-ol, 2-Pentylfuran, 2-Nonenal, Nonanal, and Octanal emerged as the major contributors to the flavor differences observed.

## 4. Discussion

The growth performance of growing–finishing pigs directly impacts both meat yield and the economic returns for livestock enterprises. In this study, replacing half of the corn with rice in the diet did not affect growth performance of growing–finishing pigs when compared to the control (CON) group, which is in line with previous studies [6]. This suggests that rice can serve as a viable alternative energy source, effectively replacing up to half of the corn in the diet without compromising growth performance in growing–finishing pigs. Additionally, the lack of negative effects on ADFI indicates that the dietary palatability was not compromised. However, a previous study has shown that substituting corn with rice improves growth performance (e.g., body weight) in weaned piglets [27]. The contrasting effects of rice on weaned piglets versus growing–finishing pigs may be attributed to differences in their physiological states, the maturity of their digestive systems, and their distinct nutritional needs at different growth stages.

Despite the lack of any adverse effects on growth performance, the rice-substituted diets significantly reduced CP, CF, and GE. These results are inconsistent with those of previous studies [5,27]. For instance, Kim et al. reported that the apparent total tract digestibility (ATTD) of GE and CP in the RICE group was superior to that of corn in weaned piglets [5]. This could be due to the immature digestive system of piglets, which is highly sensitive to anti-nutritional factors and complex starch structures in corn, giving rice starch a significant advantage in terms of digestibility. More notably, our result also differs from the findings of Kim et al. [6], who observed no differences in nutrient digestibility between corn and brown rice in growing–finishing pigs. This contradiction highlights a critical experimental variable: the physical form of the rice used. In our study, we utilized paddy rice (unhulled rice), which includes the fibrous hull and bran layers that are rich in cellulose, hemicellulose, and other anti-nutritional factors. In contrast, Kim et al. used brown rice, from which the hull has been removed. The presence of the indigestible hull in our paddy rice diet directly explains the observed decrease in apparent digestibility of CF and GE, and likely contributed to the reduced CP digestibility due to the physical encapsulation of nutrients. However, the depressed apparent digestibility of CP, CF, and GE does not necessarily imply a reduced net energy value. Rather, it likely reflects a shift in the site of nutrient digestion. Components like resistant starch in rice may bypass foregut absorption but are subsequently fermented in the hindgut, where microbial fermentation produces volatile fatty acids that provide metabolic energy. This process likely compensates for the lower pre-cecal digestibility, helping to maintain growth performance. Thus, despite differences in apparent digestibility, rice can be considered a functionally equivalent alternative to corn in growing–finishing pig diets.

The relationship between gut microbiota and host growth, development, and health has been extensively studied in recent years [28,29]. Dietary composition is crucial for shaping the composition and structure of the gut microbiota [30]. In pigs, levels of dietary protein, fiber, and fat have been shown to influence the intestinal microbiota, which in turn affects growth performance, feed utilization, skeletal muscle development, fat deposition, and immune responses [31]. Additionally, dietary enzyme additives have been reported to alter gut microbiota composition, thus improving gut health and growth performance [32]. In our study, exogenous cellulase supplementation increased the Shannon and Chao1 diversity indices. However, Long et al. [33] reported no significant effect of cellulase supplementation on alpha diversity. This variation may be explained by differences in cellulase type, dosage, and the cereal grain composition of the basal diets. Specifically, our cellulase (20,000 IU/g) was geared toward degrading rice’s crude fiber to produce available nutrients for gut microbes, whereas Long et al. used Accellerase 1000 (a complex cellulase preparation) that could not fully break down rapeseed meal’s cross-linked fiber-pectin-lignin structure, leading to distinct impacts on microbial alpha diversity. The predominant bacterial communities at the phylum level were Firmicutes and Bacteroidota, which aligns with previous reports on growing–finishing pigs [34]. Notably, exogenous cellulase supplementation further increased the relative abundance of Spirochaetota, a phylum rich in fibrolytic bacteria. Many Spirochaetota members, particularly *Treponema* species commonly found in the porcine gut, are important for breaking down complex plant cell wall polysaccharides like cellulose, hemicellulose, and pectin [35]. In addition, dietary rice substitution significantly enhanced the abundance of Firmicutes and reduced the abundance of Bacteroidota. This result is consistent with the findings of Lee et al. [36] in weaned piglets. Research has shown that the relative abundances of Firmicutes and Bacteroidetes are closely linked to fat deposition in pigs [37]. An increase in the relative abundance of *Lactobacillus* at the genus level was observed in pigs fed the rice diet. *Lactobacillus* play a crucial role in intestinal health improvement and the enhancement of fat deposition [34]. For instance, *Lactobacillus johnsonii*-derived leucine has been shown to facilitate fatty acid uptake and deposition through *CD36* [34], while *Lactobacillus reuteri* can regulate muscle fatty acid composition via the SLC22A5-mediated carnitine system [38]. Importantly, we observed an increase in IMF content in the RICE group, which may be related to the observed rise in *Lactobacillus* abundance. However, the precise underlying mechanism remains to be further investigated.

Extensive evidence indicates that the gut microbiota orchestrates host growth, development, and metabolism via the production of diverse metabolites [39]. Specifically, *Lactobacillus johnsonii* has been shown to enhance triglyceride content in cell-free supernatants cultured from IPECJ-2 cells, which promotes intestinal lipid uptake and accumulation [34]. Our previous study demonstrated that microbiota-produced succinate contributes to the development of diarrhea in weaned piglets [40]. In the present study, we observed a positive correlation between *Papillibacter* and taurochenodeoxycholic acid (TCDCA). This association raises the possibility that *Papillibacter* may interact with host lipid digestion processes, as TCDCA is a primary bile acid central to fat emulsification and absorption [41]. A potential mechanistic link may involve either the direct biotransformation of TCDCA by *Papillibacter* itself, or an indirect mechanism whereby *Papillibacter* modulates the host’s regulation of bile acid synthesis. The specific mechanism still needs further exploration. These findings strongly suggest that *Papillibacter* is involved, either directly or indirectly, in the regulation of host lipid metabolism and IMF deposition. Furthermore, *Papillibacter* is known to primarily produce acetate during fermentation [42]. Acetate serves as a key precursor for fatty acid synthesis and is transported through the bloodstream to muscle tissue, where it contributes to IMF deposition [43]. In our study, we observed a significant increase in *Papillibacter* abundance in the RICE group, and a negative correlation between *Papillibacter* and several plant secondary metabolites, including Zygadenine, Carpaine, and Rhodioloside E. This correlation suggests that *Papillibacter* might be involved in the metabolism of these plant-derived compounds, potentially playing a role in adapting to dietary changes. However, this functional inference requires direct experimental validation. Notably, these metabolites have been associated with negative regulation of fat deposition. For example, Wen et al. reported that Rhodioloside E alleviates hepatic lipid accumulation induced by a high-fat diet [44]. Consistent with this, our results demonstrated a significant negative correlation between these metabolites and IMF content, further supporting their role as inhibitors of IMF deposition. Importantly, we observed a marked positive correlation between IMF content and *Papillibacter* abundance. These findings suggest that *Papillibacter*, enriched by the rice-based diet, promotes IMF deposition, at least in part by degrading specific plant-derived metabolites. Therefore, *Papillibacter* can be considered a potential functional microorganism for improving meat quality.

Meat quality is defined by a set of properties including appearance, texture, juiciness, tenderness, flavor, and nutritional value, where aroma is a primary component of the meat flavor. To date, over 1000 volatile compounds have been reported in meat, with aldehydes, alcohols, esters, and ketones playing significant roles in shaping the overall flavor profile [45]. Studies have shown that variations in volatile compounds among different pig breeds contribute to distinct meat quality characteristics [46]. Additionally, the composition of the diet serves to shape the volatile compound profile in meat, thereby affecting its quality [47]. We detected a total of 170 volatile compounds across all samples, confirming that aldehydes, alcohols, and esters are key contributors to the flavor profile of pork, consistent with previous reports [48]. Notably, we observed an increase in the levels of various aldehydes, such as nonanal and octanal, in the RASE group. Aldehydes, known for their low odor thresholds, are often associated with fatty and grassy aromas and are primary products of fatty acid oxidative degradation in meat [49]. For instance, hexanal and heptanal are produced through the oxidative breakdown of linoleic acid and arachidonic acid, while oleic acid can be oxidized to form octanal and nonanal [50]. Lipids are the core precursors of flavor compounds such as aldehydes and alcohols, and the gut microbiota can regulate lipid synthesis and decomposition through metabolites. This study indicates that *Papillibacter* may alleviate the inhibitory effects of metabolites like Zygadenine and Carpaine on fat deposition, thereby influencing IMF deposition. Since IMF accumulation is fundamentally the deposition of fatty acids [51], *Papillibacter* could potentially modulate fatty acid composition by regulating the levels of these metabolites. Additionally, fermentation by *Papillibacter* can produce acetate, a precursor for fatty acid synthesis, which may elevate the levels of unsaturated fatty acids such as oleic and linoleic acids in muscle [42]. Thus, the composition of flavor compounds is likely related to the remodeling of lipid metabolism mediated by gut *Papillibacter*. These results indicate that the combined use of rice and cellulase in the diet influences lipid metabolism and composition in the muscle, contributing to distinct flavor characteristics. Moreover, 1-octen-3-ol, derived from the catabolism of linoleic and arachidonic acids [52], was identified as a key determinant of pork flavor, owing to its pronounced mushroom-like scent and low detection threshold [53]. Moreover, the odor profile of 3-octanone is described as mushroom-like, musty, and slightly cheesy. Therefore, the significantly increased 1-octen-3-ol in the ASE group and elevated 3-octanone in the RICE group could serve as potential markers for specific dietary treatments.

## 5. Conclusions

In conclusion, partial replacement of corn with rice in the diet of growing–finishing pigs may potentially facilitate IMF deposition by enriching *Papillibacter* and modulating host metabolism, including the reduction in metabolites such as Zygadenine, Rhodioloside E, and Carpaine, without adversely affecting growth performance. Additionally, the combined dietary intervention of rice and cellulase increased the levels of key volatile compounds, including 2-pentylfuran, 2-nonenal, nonanal, and octanal, contributing to an improved pork flavor profile. These findings suggest that using rice alone or in combination with cellulase can serve as an effective strategy to enhance both the nutritional and sensory qualities of pork.

## Figures and Tables

**Table 1:** Ingredients and nutrient composition of the diets.

Ingredients, %	CON	RICE	ASE	RASE
Corn	70.00	35.00	70.00	35.00
Paddy rice	0.00	35.00	0.00	35.00
Flour	2.00	2.00	2.00	2.00
Wheat bran	8.00	6.60	8.00	6.60
Soybean meal	13.00	14.00	13.00	14.00
Soybean oil	1.00	4.00	1.00	4.00
Dicalcium phosphate	0.80	0.65	0.80	0.65
Stone powder	1.20	1.30	1.20	1.30
Salt	0.30	0.30	0.30	0.30
L-Lys sulfate 78%	0.20	0.15	0.20	0.15
Thr	0.00	0.02	0.00	0.02
Complex multidimensional ^1^	0.03	0.03	0.03	0.03
Trace element premix ^2^	0.10	0.10	0.10	0.10
Antifungal agents	0.20	0.20	0.20	0.20
Antioxidant	0.03	0.03	0.03	0.03
Choline chloride 50%	0.10	0.10	0.10	0.10
Bentonite	3.04	0.52	3.02	0.50
Cellulase	0.00	0.00	0.02	0.02
Total	100.00	100.00	100.00	100.00
**Calculated value, %**				
NE, Mcal/kg	3.16	3.17	3.16	3.17
CP, %	13.87	13.88	13.87	13.88
Ca, %	0.73	0.73	0.73	0.73
P, %	0.61	0.58	0.61	0.58
Available phosphorus, %	0.26	0.26	0.26	0.26
SID-Lys, %	0.64	0.64	0.64	0.64
SID-Met, %	0.20	0.21	0.20	0.21
SID-Thr, %	0.40	0.40	0.40	0.40
SID-Trp, %	0.12	0.13	0.12	0.13
**Analyzed value, %**				

^1^ Provided for per kilogram of diet: vitamin A, 32.5 million IU; vitamin B_1_, 10 g; vitamin B_2_, 25 g; vitamin B_6_, 15 g; vitamin B_12_, 100 mg; vitamin D_3_, 10 million IU; vitamin E, 80 g; vitamin K_3_, 10 g; nicotinamide, 120 g; D-pantothenic acid, 70 g; folic acid, 5 g; D-biotin, 500 mg. ^2^ Provided for per kilogram of diet: FeSO_4_·H_2_O, 348.97 g; CuSO_4_·5H_2_O, 48.83 g; ZnSO_4_·H_2_O, 193.21 g; MnSO_4_·H_2_O, 84.51 g; Ca (IO_3_)_2_·H_2_O, 0.768 g; Na_2_SeO_3_·5H_2_O, 1.18 g; Fe, 115 g; Cu, 12.5 g; Zn, 70 g; Mn, 27.5 g; I, 0.5 g; Se, 0.35 g.

**Table 2:** Effects of rice and cellulase on growth performance in growing–finishing pigs.

Item	Corn		Rice		*p* Value		
	CON	ASE	RICE	RASE	Diet	Enzyme	Interaction
IBW, kg	68.01 ± 1.24	68.09 ± 1.86	68.01 ± 1.20	68.01 ± 2.28	0.949	0.957	0.957
FBW, kg	134.0 ± 5.8	131.3 ± 3.4	134.3 ± 1.7	133.4 ± 4.5	0.321	0.460	0.812
ADG, kg	1.03 ± 0.10	1.00 ± 0.06	1.05 ± 0.03	1.04 ± 0.06	0.318	0.455	0.799
ADFI, kg	3.10 ± 0.15	3.08 ± 0.18	3.03 ± 0.45	2.99 ± 0.13	0.460	0.780	0.966
F/G	3.03 ± 0.23	3.08 ± 0.26	2.88 ± 0.40	2.89 ± 0.16	0.146	0.785	0.869

IBW = initial body weight; FBW = final body weight; ADG = average daily gain; ADFI = average daily feed intake; F/G = feed conversion ratio. CON = basal diet; ASE = basal diet supplemented with 20,000 IU/g cellulase; RICE = basal diet in which 50% of the corn was replaced by paddy rice; RASE = basal diet with 50% corn replaced by paddy rice and supplemented with cellulase.

**Table 3:** Effects of rice and cellulase on apparent digestibility in growing–finishing pigs.

Item	Corn		Rice		*p* Value		
	CON	ASE	RICE	RASE	Diet	Enzyme	Interaction
CP, %	83.64 ± 1.46 ^a^	82.77 ± 3.65 ^a^	73.08 ± 6.64 ^b^	76.83 ± 2.02 ^b^	<0.001	0.0434	0.214
CF, %	66.57 ± 7.70 ^a^	62.44 ± 6.80 ^ab^	59.49 ± 7.74 ^ab^	56.03 ± 5.46 ^b^	0.028	0.198	0.906
GE, %	85.58 ± 1.69 ^a^	87.50 ± 1.85 ^a^	78.96 ± 3.69 ^b^	79.51 ± 5.00 ^b^	<0.001	0.382	0.632

CP = Crude protein; CF = crude fiber; GE = Gross energy; CON = basal diet; ASE = basal diet supplemented with 20,000 IU/g cellulase; RICE = basal diet in which 50% of the corn was replaced by paddy rice; RASE = basal diet with 50% corn replaced by paddy rice and supplemented with cellulase. ^a, b^ Different superscript letters in the same row indicate significant differences (*p* < 0.05).

**Table 4:** Effects of rice and cellulase on serum biochemistry in growing–finishing pigs.

Item	Corn		Rice		*p* Value		
	CON	ASE	RICE	RASE	Diet	Enzyme	Interaction
ALT, U/L	59.0 ± 22.5 ^a^	39.8 ± 8.1 ^c^	47.5 ± 8.8 ^b^	49.5 ± 17.5 ^ab^	0.768	0.007	0.001
AST, U/L	52.1 ± 18.2 ^a^	33.0 ± 9.5 ^b^	42.4 ± 8.9 ^a^	55.3 ± 44.1 ^ab^	0.174	0.506	0.001
CK U/L	3717 ± 1796 ^a^	2581 ± 1373 ^b^	3773 ± 1638 ^a^	4074 ± 3202 ^ab^	0.059	0.305	0.079
LACT, mmol/L	13.6 ± 3.9 ^a^	10.7 ± 1.9 ^b^	14.34 ± 3.4 ^a^	12.5 ± 2.3 ^a^	0.031	0.000	0.414

ALT = alanine aminotransferase; AST = aspartate aminotransferase; CK = creatine kinase; LACT = lactate. CON = basal diet; ASE = basal diet supplemented with 20,000 IU/g cellulase; RICE = basal diet in which 50% of the corn was replaced by paddy rice; RASE = basal diet with 50% corn replaced by paddy rice and supplemented with cellulase. ^a, b, c^ Different superscript letters in the same row indicate significant differences (*p* < 0.05).

**Table 5:** Effects of rice and cellulase on pork quality in growing–finishing pigs.

Item	Corn		Rice		*p* Value		
	CON	ASE	RICE	RASE	Diet	Enzyme	Interaction
BFT, mm	27.25 ± 7.27	25.04 ± 3.68	28.58 ± 4.18	26.51 ± 1.53	0.469	0.273	0.971
DL, %	3.32 ± 0.80	3.31 ± 0.07	2.38 ± 0.10	2.49 ± 0.10	0.096	0.925	0.914
pH_45_	5.92 ± 0.41	6.16 ± 0.12	6.11 ± 0.11	6.09 ± 0.15	0.538	0.259	0.189
pH_24_	5.35 ± 0.14	5.31 ± 0.13	5.28 ± 0.12	5.20 ± 0.07	0.065	0.227	0.727
Color							
L	48.81 ± 2.81	53.56 ± 4.43	54.27 ± 5.10	55.33 ± 4.46	0.123	0.207	0.415
a	17.84 ± 2.09	19.21 ± 3.88	19.67 ± 2.07	19.34 ± 2.77	0.488	0.711	0.546
b	4.99 ± 1.51	7.10 ± 0.68	6.93 ± 2.11	6.77 ± 1.19	0.311	0.226	0.161
IMF, %	2.69 ± 1.07 ^a^	2.34 ± 0.56 ^a^	4.25 ± 1.34 ^b^	3.37 ± 1.02 ^a,b^	0.006	0.162	0.536

BFT = backfat thickness; DL = drip loss; IMF = Intramuscular fat. CON = basal diet; ASE = basal diet supplemented with 20,000 IU/g cellulase; RICE = basal diet in which 50% of the corn was replaced by paddy rice; RASE = basal diet with 50% corn replaced by paddy rice and supplemented with cellulase. ^a, b^ Different superscript letters in the same row indicate significant differences (*p* < 0.05).

**Table 6:** Effects of rice and cellulase on lipid metabolism-related genes in *longissimus dorsi* muscle of growing–finishing pigs.

Item	Corn		Rice		*p* Value		
	CON	ASE	RICE	RASE	Diet	Enzyme	Interaction
*PPARα*	1.12 ± 0.25 ^a^	0.56 ± 0.27 ^ab^	0.49 ± 0.10 ^b^	0.21 ± 0.47 ^b^	0.024	0.051	0.628
*SREBP1*	1.02 ± 0.99	0.98 ± 0.11	0.93 ± 0.15	0.72 ± 0.15	0.196	0.347	0.533
*SREBP2*	1.13 ± 0.28 ^a^	0.70 ± 0.12 ^ab^	0.84 ± 0.11 ^ab^	0.49 ± 0.03 ^b^	0.133	0.027	0.808
*LXRβ*	1.09 ± 0.19	1.18 ± 0.15	1.03 ± 0.07	0.86 ± 0.09	0.172	0.759	0.337
*CD36*	1.02 ± 0.25	1.04 ± 0.64	1.61 ± 0.37	1.06 ± 0.11	0.092	0.142	0.115
*DGAT1*	1.04 ± 0.14 ^a^	0.69 ± 0.10 ^ab^	0.96 ± 0.14 ^a^	0.55 ± 0.38 ^b^	0.330	0.003	0.749
*DGAT2*	1.42 ± 0.56	1.13 ± 0.37	0.97 ± 0.15	0.50 ± 0.10	0.134	0.287	0.817

^a, b^ Different superscript letters in the same row indicate significant differences (*p* < 0.05).

## Data Availability

The original contributions presented in this study are included in the article/Supplementary Materials. Further inquiries can be directed to the corresponding authors.

## Supplementary Materials

The following supporting information can be downloaded at: https://www.mdpi.com/article/10.3390/ani16010012/s1, Table S1: Primers for real-time PCR gene expression analysis;. Table S2: Relative abundance of top 10 phyla in fecal microbiota of growing-finishing pigs; Table S3: Relative abundance of top 10 genera in fecal microbiota of growing-finishing pigs; Table S4: The relative contents and types of volatile compound in Longissimus dorsi muscle; Table S5: Relative Odor Activity Value of Volatile Compounds.
