# Supplementary material for: Effect of Replacing Corn with Rice on Growth Performance, Meat Quality, Gut Microbiota and Metabolites in Growing–Finishing Pigs
Source: Animals (Basel). 2025 Dec 19;16(1):12. doi: 10.3390/ani16010012 (PMC12784687; doi:10.3390/ani16010012)
Supplement: Supplementary file 1 [file animals-16-00012-s001.zip › animals-4004501-supplementary.pdf]

Supplementary Table S1. Primers for real-time PCR gene expression analysis

Gene	Forward sequence (5′-3′)	Reverse sequence (3′-5′)
β-Actin	GTCCACCTTCCAGCAGATGT	GAAAGGGTGTAAAACGCAGC
PPARα	GTGCAGCCTCAGCCAAGTT	TGGGGAGAGAGGACAGATGG
SREBP1	ACTTTTCCTTAACGTGGGCCT	TGAGCTGGAGCATGTCTTCG
SREBP2	GCTGTCGGGTGTCATGGG	ACAAACTGTAGCATCTCGTCGAT
LXRβ	GCTACAACCACGAGACAGAAT	GGCGATAAGCAAGGCATACT
CD36	TGGGTTAAAACAGGCACGGA	ACTGTGTGGGTCTCAGGGTC
DGAT1	AACCTGACCTACCGCGATCT	GGAAGCGGGAAAAGTTGAGC
DGAT2	CGCTCTACTTCACTTGGCTG	TGGTGAGCAGGTTGTGTGTC

Supplementary Table S2 Relative abundance of top 10 phyla in fecal microbiota of growing-finishing pigs

Item	Corn		Rice		P value		
	CON	ASE	RICE	RASE	Diet	Enzyme	Interaction
Firmicutes	83.57±4.64[a]	80.77±11.26[a]	89.61±3.57[a]	87.50±5.30[a]	0.034	0.390	0.908
Bacteroidota	10.35±7.24[a]	11.16±8.27[a]	4.57±3.20[a]	4.89±3.07[a]	0.022	0.818	0.921
Spirochaetota	0.89±1.40[a]	3.70±4.31[a]	0.78±0.82[a]	3.81±3.13[a]	0.997	0.018	0.923
Euryarchaeota	3.53±3.42	2.70±2.30	3.00±1.31	1.69±1.70	0.423	0.273	0.800
Proteobacteria	0.33±0.30	0.23±0.22	0.71±1.42	0.63±0.76	0.258	0.791	0.986
Actinobacteriota	1.02±0.84	0.63±0.33	0.67±0.34	0.76±0.38	0.594	0.489	0.271
Patescibacteria	0.05±0.06	0.21±0.48	0.07±0.08	0.07±0.10	0.568	0.421	0.444
Verrucomicrobiota	0.10±0.09	0.33±0.34	0.27±0.19	0.34±0.36	0.401	0.180	0.486
Cyanobacteria	0.06±0.06[a]	0.07±0.05[a]	0.19±0.13[ab]	0.21±0.13[a]	0.003	0.650	0.809
Campylobacterota	0.01±0.02	0.06±0.09	0.01±0.01	0.03±0.06	0.395	0.120	0.512

Supplementary Table S3 Relative abundance of top 10 genera in fecal microbiota of growing-finishing pigs

Item	Corn		Rice		P value		
	CON	ASE	RICE	RASE	Diet	Enzyme	Interaction
Solibacillus	0.00±0.00	0.01±0.02	8.04±19.67	0.00±0.00	0.329	0.329	0.328
Streptococcus	26.28±14.94	10.91±10.07	16.61±11.72	20.17±12.17	0.969	0.256	0.075
Terrisporobacter	9.54±5.84	14.45±15.09	7.26±5.41	7.82±2.94	0.222	0.448	0.545
Lactobacillus	1.65±1.35[a]	1.95±3.41[a]	9.82±12.84[a]	10.28±12.32[a]	0.038	0.919	0.982
Parabacteroides	2.97±6.92	1.02±1.26	0.41±0.45	0.15±0.13	0.248	0.450	0.563
Lachnospiraceae _XPB1014_group	3.31±3.93	2.74±2.69	4.54±3.80	5.80±5.98	0.232	0.848	0.608
UCG_002	2.38±1.79[a]	6.05±3.12[a]	4.31±5.28[a]	2.41±1.16[a]	0.524	0.509	0.048
Clostridium _sensu_stricto_1	6.44±4.74	5.32±3.21	4.37±3.26	3.82±1.35	0.210	0.551	0.836
NK4A214_group	1.71±1.21	2.14±0.82	4.56±4.14	2.14±0.93	0.137	0.291	0.134
Treponema	0.89±1.39[a]	3.68±4.29[a]	0.77±0.81[a]	3.79±3.12[a]	0.999	0.018	0.919

Supplementary Table S4 The relative contents and types of volatile compound in *Longissimus dorsi* muscle

Volatile Class I	CON (%)	ASE (%)	RICE (%)	RASE (%)
Aldehydes	61.2916	60.1513	58.9135	60.431
Alcohols	6.1335	7.9841	7.7993	9.7635
Esters	9.8239	9.4579	12.288	6.2572
Ketones	5.2191	6.4821	5.4441	8.0923
Hydrocarbons	5.9221	5.5622	5.573	4.9655
Terpenoids	0.789	0.6501	0.6236	0.6384
Unknown	4.3771	2.8186	2.4232	1.9885
Phenols	0.5179	0.4794	0.4095	0.3529
Acids	1.8124	2.2039	1.9097	2.5563
Organoheterocyclic compounds	2.539	2.6698	3.0003	3.9494
Organicnitrogen compounds	1.1748	1.1994	1.276	0.7068
Phenol ethers	0.1087	0.0907	0.079	0.0724
Organosulfur compounds	0.1293	0.118	0.1309	0.1198
Haloalkanes	0.1615	0.1325	0.13	0.106

Supplementary Table S5 Relative Odor Activity Value of Volatile Compounds

Metabolites	Odor Threshold	CC(ROAV)	CE(ROAV)	RC(ROAV)	RE(ROAV)
ASE vs. CON					
1-Octen-3-Ol	0.007	1.9072	2.0398	1.9248	1.5842
Cis-5-Octen-1-Ol	0.002~0.003	1.5904	1.6351	1.2854	0.9376
RICE vs. CON					
3-Octanone	1	0.0014	0.0014	0.0016	0.0009
RASE vs. ASE					
2-Pentylfuran	0.0048	1.7288	1.3149	1.608	1.6955
2-Nonenal	0.000065	18.8601	17.6863	19.1622	22.3652
Nonanal	0.015	1.4207	0.7896	0.7197	0.9193
Octanal	0.0007	13.2594	9.2975	7.8785	11.6215
Toluene	0.14	0.17	0.1184	0.1239	0.0632
RASE vs. RICE					
2-Decenal	0.3~0.4	0.0035	0.0038	0.0031	0.0056
Nonanal	0.015	1.4207	0.7896	0.7197	0.9193
Octanal	0.0007	13.2594	9.2975	7.8785	11.6215
Heptanal	0.01	0.5753	0.4601	0.3741	0.4869
1-Heptanol	0.2	0.0163	0.0164	0.0132	0.0181
2-Octenal	3	0.0005	0.0005	0.0004	0.0006
